# Effects of logging, hunting, and forest fragment size on physiological stress levels of two sympatric ateline primates in Colombia

**DOI:** 10.1093/conphys/cot031

**Published:** 2013-11-21

**Authors:** Rebecca Rimbach, Andrés Link, Michael Heistermann, Carolina Gómez-Posada, Nelson Galvis, Eckhard W. Heymann

**Affiliations:** 1 Behavioral Ecology and Sociobiology Unit, German Primate Center, Kellnerweg 4, 37077 Göttingen, Germany; 2 Fundación Proyecto Primates, Cra. 11a No. 91-55, Bogotá, Colombia; 3 Departamento de Ciencias Biológicas, Universidad de Los Andes, Cra. 1 No. 18a-12, Bogotá, Colombia; 4 Endocrinology Laboratory, German Primate Center, Kellnerweg 4, 37077 Göttingen, Germany; 5 Department of Biology, University of Washington, 106 Kincaid, Seattle, WA 98195, USA

**Keywords:** *Alouatta seniculus*, *Ateles hybridus*, faecal glucocorticoid metabolites, habitat fragmentation, hunting, logging

## Abstract

Habitat fragmentation and anthropogenic disturbances are of major concern to the conservation of endangered species because of their potentially negative impact on animal populations. Both processes can impose physiological stress (i.e. increased glucocorticoid output) on animals, and chronically elevated stress levels can have detrimental effects on the long-term viability of animal populations. Here, we investigated the effect of fragment size and human impact (logging and hunting pressure) on glucocorticoid levels of two sympatric Neotropical primates, the red howler monkey (*Alouatta seniculus*) and the critically endangered brown spider monkey (*Ateles hybridus*). These two species have been reported to contrast strongly in their ability to cope with anthropogenic disturbances. We collected faecal samples from eight spider monkey groups and 31 howler monkey groups, living in seven and 10 different forest fragments in Colombia, respectively. We measured faecal glucocorticoid metabolite (FGCM) levels in both species using previously validated methods. Surprisingly, fragment size did not influence FGCM levels in either species. Spider monkeys showed elevated FGCMs in fragments with the highest level of human impact, whereas we did not find this effect in howler monkeys. This suggests that the two species differ in their physiological responsiveness to anthropogenic changes, further emphasizing why brown spider monkeys are at higher extinction risk than red howler monkeys. If these anthropogenic disturbances persist in the long term, elevated FGCM levels can potentially lead to a state of chronic stress, which might limit the future viability of populations. We propose that FGCM measurements should be used as a tool to monitor populations living in disturbed areas and to assess the success of conservation strategies, such as corridors connecting forest fragments.

## Abstract (precis)

This study provides evidence that hunting and logging can impose stress on animals. Spider monkeys showed elevated fecal glucocorticoid metabolite (FGCM) levels in forest fragments with high levels of human impact, whereas howler monkeys did not. Glucocorticoid measurements can be a useful tool to monitor wildlife populations in disturbed areas.

## Introduction

Habitat loss, habitat fragmentation, and anthropogenic disturbances that accompany these processes (e.g. logging, increased hunting pressure) are of major concern to the conservation of endangered species due to their role in population declines (Peres, 2001; Fahrig, 2003). Currently, numerous species from all vertebrate groups are threatened with extinction (IUCN, 2012). The pervasive process of anthropogenic disturbances of natural ecosystems (Hannah *et al.*, 1995; Foley *et al.*, 2005) emphasizes the need to understand the proximate effects that these alterations have on the health and survival of animal populations. Generally, taxa with narrow dietary niches and that occupy few habitat types are at greater extinction risk than taxa that have broader niches and occupy several habitat types (Harcourt *et al.*, 2002). Species respond to fragmentation and disturbances differently depending on factors such as life history, geographical range, ecological niche, and dispersal ability (Purvis *et al.*, 2000; Henle *et al.*, 2004; Cardillo *et al.*, 2005). Some species manage to adjust aspects of their behaviour and social system (Menon and Poirier, 1996; Sumner *et al.*, 1999; González-Solís *et al.*, 2001; Blumstein *et al.*, 2005; Umapathy *et al.*, 2011), whereas species that cannot adjust face local extinction (Cosson *et al.*, 1999; Peres, 2001).

The role that changes in physiological parameters play in population declines and, ultimately, species extinction is not yet well understood. Glucocorticoids (GCs; cortisol and corticosterone, depending on the species) are the frontline hormones of the vertebrate stress response (Sapolsky *et al.*, 2000) and potentially play an important role. Many different stimuli (e.g. predators, aggression from conspecifics, food deprivation) can elicit a stress response that triggers the release of GCs. Such elevations of GC levels are adaptive and important for the survival of the individual (Boonstra, 2005; Monclús *et al.*, 2005). Glucocorticoids mobilize readily available energy that can be used to respond to the stimulus that triggered the release of GCs (Selye, 1956; Breazile, 1987; Stratakis and Chrousos, 1995). During the stress response, other energetically demanding activities, such as digestion and reproduction, are suppressed (Wingfield *et al.*, 1998; Landys *et al.*, 2006). Hence, long-term elevations of GCs can have deleterious effects on reproduction, growth, and immune system activity (Pickering *et al.*, 1991; Cyr and Romero, 2007; Charbonnel *et al.*, 2008; French *et al.*, 2010; Setchell *et al.*, 2010). Anthropogenic disturbances have been associated with GC elevations in many vertebrate taxa (amphibians: Homan *et al.*, 2003; Janin *et al.*, 2011; reptiles: French *et al.*, 2010; birds: Lucas *et al.*, 2006; Wasser *et al.*, 1997; and mammals: Martínez-Mota *et al.*, 2007; Gobush *et al.*, 2008; Rangel-Negrín *et al.*, 2009; Jaimez *et al.*, 2012; Dunn *et al.*, 2013). These GC elevations have been linked to negative effects on reproduction and immune system activity (e.g. Ellenberg *et al.*, 2007; French *et al.*, 2010), suggesting that elevated stress levels caused by human influence can directly affect individual health and, ultimately, population viability.

We studied two primate species that occur sympatrically in Colombia, namely brown spider monkeys (*Ateles hybridus*) and red howler monkeys (*Alouatta seniculus*), to investigate whether and how anthropogenic disturbances influence their GC output. The two genera *Ateles* and *Alouatta* have been reported to contrast strongly in their ability to cope with anthropogenic disturbances (Bernstein *et al.*, 1976; Bicca-Marques, 2003; Michalski and Peres, 2005). Brown spider monkeys are endemic to Colombia and Venezuela, and are considered to be one of the 25 most endangered primate species worldwide due to severe habitat loss and high hunting pressure (Mittermeier *et al.*, 2012). Habitat fragmentation, slow reproductive cycles, large area requirements, and their dietary niche (for review, see Di Fiore *et al.*, 2010) make this species vulnerable to anthropogenic disturbances. Moreover, spider monkeys exhibit flexible grouping patterns (fission–fusion dynamics) that are likely to reduce intra-group feeding competition and enable them to cope with changes in the availability of ripe fruit, their preferred food resources (e.g. Klein and Klein, 1977; Symington, 1988). Accordingly, the confinement to small fragments might reduce the flexibility of their grouping patterns and thereby lower their potential to minimize competition, which might cause increased physiological stress.

In contrast, red howler monkeys seem less vulnerable to anthropogenic disturbances and are able to persist even in extremely small fragments (Crockett, 1998; Lopez *et al.*, 2005; Michalski and Peres, 2005). They have a broad distribution range, occupy a wide array of ecosystems, and are currently not threatened with extinction (Boubli *et al.*, 2008). Typically, howler monkeys require much smaller home ranges than spider monkeys (reviewed by Di Fiore *et al.*, 2010). They from cohesive groups (Neville, 1972; for review, see Di Fiore and Campbell, 2007) and have a mainly folivorous but flexible diet (Milton, 1980; Julliot and Sabatier, 1993; Bicca-Marques, 2003).

The proximate mechanisms leading to such species-specific differences in the ability to cope with a changing environment have not yet been investigated. Therefore, the aim of this study was to determine how fragment size and level of human impact influence physiological stress levels, measured through faecal glucocorticoid metabolites (FGCMs), in both species. We collected faecal samples from both species in forest fragments that differed in size and level of human impact (hunting and logging activities). We predicted that FGCM levels would increase with both, decreasing fragment size (as a proxy for fragmentation intensity) and increasing level of human impact. Due to differences in diet, area requirements, and adaptation capabilities, we predicted that red howler monkeys would generally react less strongly than brown spider monkeys to these disturbances, and that they would show no elevation in GC levels or a less pronounced elevation compared with brown spider monkeys.

## Materials and methods

### Study sites

Between April 2010 and April 2012, we collected faecal samples from eight spider monkey and 31 howler monkey groups, living in seven and 10 different fragments, respectively. Fragments differed in size and human impact (Table 1). Four fragments (San Juan, Quinchas, Jamaica, and Juntas) are long-term study sites, and study groups have been habituated to human observers previously (Aldana *et al.*, 2008; Gómez-Posada *et al.*, 2010; Link *et al.*, 2010). We visited all other fragments only for faecal sample collection purposes (for 2–3 weeks). Nine of the study fragments were occupied by both species and the other four (Jamaica, Juntas, LGPM, and Cienaga) were occupied only by howler monkeys. Although most fragments contained both species, sometimes faecal sample collection was feasible only for one. We determined the level of current human impact through observations (e.g. recently cut tree stumps, presence of hunting dogs in fragments, pet primates at farms) and surveys in which we interviewed farm owners and workers. We classified fragments without current human hunting activities and absence of recent logging activity (≤2 months) as level 0, fragments with either hunting or logging activity as level 1, and fragments with both hunting and ongoing logging activity as level 2.

**Table 1:** Number of faecal samples collected for each species in the different forest fragments (*n* = 13), which varied in size and level of human impact

			No. of samples (groups)		
Fragment	Size (ha)	Human impact	*Ateles hybridus*	*Alouatta seniculus*	Location
San Juan	65	0	411 (2)	289 (17)	6° 43′N, 74° 09′W
San Juan3	75	0	10 (1)	5 (1)	6° 43′N, 74° 07′W
LGPM	100	0	—	9 (1)	6° 41′N, 74° 09′W
Quinchas	250	0	46 (1)	21 (3)	6° 02′N, 74° 16′W
Terra Firme1	500	0	3 (1)	—	6° 41′N, 74° 08′W
LGPM2	500	0	—	4 (1)	6° 41′N, 74° 09′W
Jamaica	4.21	1	—	12 (1)	4° 23′N, 75° 48′W
Juntas	25.5	1	—	13 (1)	4° 25′N, 75° 47′W
India	500	1	3 (1)	—	6° 15′N, 74° 07′W
Cienaga	50	2	—	6 (2)	6° 42′N, 74° 08′W
Campo Capote	250	2	3 (1)	5 (1)	6° 34′N, 73° 51′W
Remedios	400	2	5 (1)	—	6° 53′N, 74° 34′W
San Juan 4	500	2	—	9 (3)	6° 41′N, 74° 07′W
Total no. of samples			481 (8)	373 (31)	

### Faecal sample collection

We collected samples from adult and subadult individuals (mean ± SD: *Ateles*, 7.9 ± 12.5 samples per individual and *Alouatta*, 2.3 ± 2.6 samples per individual) and, for every sample, noted sex and age-class, female reproductive state (when identifiable), collection time, and date. The time lag of GC metabolite excretion in faeces is ∼24 h in *A. hybridus* and ∼46 h in *A. seniculus* (Rimbach *et al.*, 2013). Thus, we avoided following unhabituated groups on 2–3 consecutive days (depending on the species) to reduce the influence of observer presence on FGCM levels. In unhabituated groups, we did not recognize individuals and, to avoid resampling of individuals, we sampled each group or subgroup (in the case of *Ateles*) only once. Faecal samples collected in the fragments ‘Jamaica’ and ‘Juntas’ were collected between May and July 2010, and stored at −20°C until extraction in December 2012. Long-term faecal sample storage at −20°C has been shown to preserve immunoreactive faecal GC metabolites reliably (Hunt and Wasser, 2003), and we are therefore confident that this storage method has not affected the FGCM measurements of these samples.

In both species, pregnancy cannot reliably be detected by observation. Thus, to determine the approximate conception date and female reproductive state at sample collection we used parturition date (habituated groups) in combination with average gestation length (*Ateles*: ∼7.5 months and *Alouatta*: ∼6.3 months; Di Fiore and Campbell, 2007). We categorized females as lactating for the time in which they nursed dependent offspring. Females that were neither pregnant nor lactating were categorized as non-pregnant, non-lactating. However, in unhabituated groups we were able to categorize females merely as either lactating or non-lactating (pregnant and cycling females). We collected comparative numbers of samples from both sexes (*A. hybridus*: 51.8% females and 48.2% males; and *A. seniculus*: 45.6% females and 54.4% males) per fragment and have no reason to assume that the collection was biased towards collecting samples from females in only certain reproductive conditions.

Before placing faecal material into the sample tube, we homogenized the faecal bolus and removed any obvious undigested matter (e.g. large seeds). We placed ∼0.5 g of faeces into a 15 ml pre-weighed polypropylene tube pre-filled with 5 ml of 96% ethanol and shook the tube until the faeces were suspended in the solvent (Shutt *et al.*, 2012; Rimbach *et al.*, 2013). We kept the samples at ambient temperatures until steroid extraction in the evening.

### Steroid extraction and faecal glucocorticoid metabolite analysis

We determined faecal wet weight by calculating the difference between the weight of the tube before (tube plus ethanol) and after sample collection (tube, ethanol, and faeces). We shook the tubes firmly for 5 min and thereafter, centrifuged the samples for 1 min using a manually operated centrifuge (Shutt *et al.*, 2012; Rimbach *et al.*, 2013). We poured off ∼2 ml of each faecal extract into 2 ml polypropylene tubes, covered them with parafilm, labelled them, and stored them at ambient temperatures (∼25°C) in a dark place. Every 2 months we transported the extracts to the Universidad de Los Andes, Bogotá and stored them at −20°C until shipment to the Endocrinology Laboratory at the German Primate Center for analysis. We showed in a previous study that storing faecal extracts in this way did not affect FGCM levels (Rimbach *et al.*, 2013).

We analysed all faecal samples using a previously validated (Rimbach *et al.*, 2013) enzyme-immunoassay for 11β-hydroxyetiocholanolone, a group-specific measurement of 5β-reduced GC metabolites (Ganswindt *et al.*, 2003) with a 3α,11β-dihydroxy structure. The enzyme-immunoassay was performed as described in detail by Heistermann *et al.* (2004). Prior to hormone measurement, we diluted extracts 1:250–1:2000 (depending on original concentration) in assay buffer and thereafter, took duplicate aliquots to assay. Intra- and inter-assay coefficients of variation of were 6.1 and 7.8%, respectively, for high-value and 7.4 and 13.0%, respectively, for low-value quality controls. All hormone concentrations are expressed as nanograms per gram faecal wet weight.

### Statistical analyses

To assess the influence of fragment size and level of human impact on FGCM levels, we used linear mixed models (LMM; Baayen, 2010). We fitted all models with the lmer function from the lme4 R-package (Bates and Maechler, 2010) in R2.15.1 (R Development Core Team 2012). We used restricted maximal likelihood methods to estimate the models, because they are robust against unequal sample sizes (Keselman *et al.*, 2001). We used two LMMs per species, and group and fragment ID were used as random factors in all models. Glucocorticoid excretion often shows a diurnal rhythm, and GC levels can be affected by a variety of potentially confounding factors (for review, see Millspaugh and Washburn, 2004; Keay *et al.*, 2006; Goymann, 2012). To account for these factors, we used sex, age, female reproductive state, group size, and sample collection time as control variables in the models (because some of these variables have been shown to affect FGCM levels in the study species; Rimbach *et al.*, 2013). We tested for interactions between fragment size and human impact, and between group size and fragment size. These were not significant (*P* ≥ 0.05) and are not included in the final models. For each study species we used two models, one using the size of each fragment and one comparing fragments with ‘control sites’. In the second set of models, we defined ‘control sites’ as fragments that were larger than a certain, species-specific, size. Due to differences in area requirements between the study species (Di Fiore *et al.*, 2010), we defined fragments larger than 50 ha as ‘control sites’ (or as a proxy for continuous forest) for red howler monkeys, whereas for brown spider monkeys we defined fragments larger than 200 ha as ‘control sites’.

We log transformed the response variable (FGCM levels) to achieve normal distribution and checked that the assumptions of normally distributed and homogeneous residuals were fulfilled by visually inspecting Q–Q plots and the residuals plotted against the fitted values for each model. To assess model stability, we ran diagnostics (dfbetas) that did not suggest the existence of influential cases. We used the function vif of the R-package car (Fox and Weisberg, 2011) applied to a standard linear model, excluding the random effects, to derive variance inflation factors (Field, 2005). We used a likelihood ratio test (R function ‘anova’) to determine the significance of the full model (all fixed and random effects) compared with the corresponding null model (only random effects). We used the functions pvals.fnc of the R-package ‘language R’ (Baayen, 2010) to determine *P* values based on Markov Chain Monte Carlo (MCMC) sampling (Baayen, 2011). All statistical tests were two tailed, and the statistical threshold was set at *P* ≤ 0.05.

## Results

**Figure 1:**
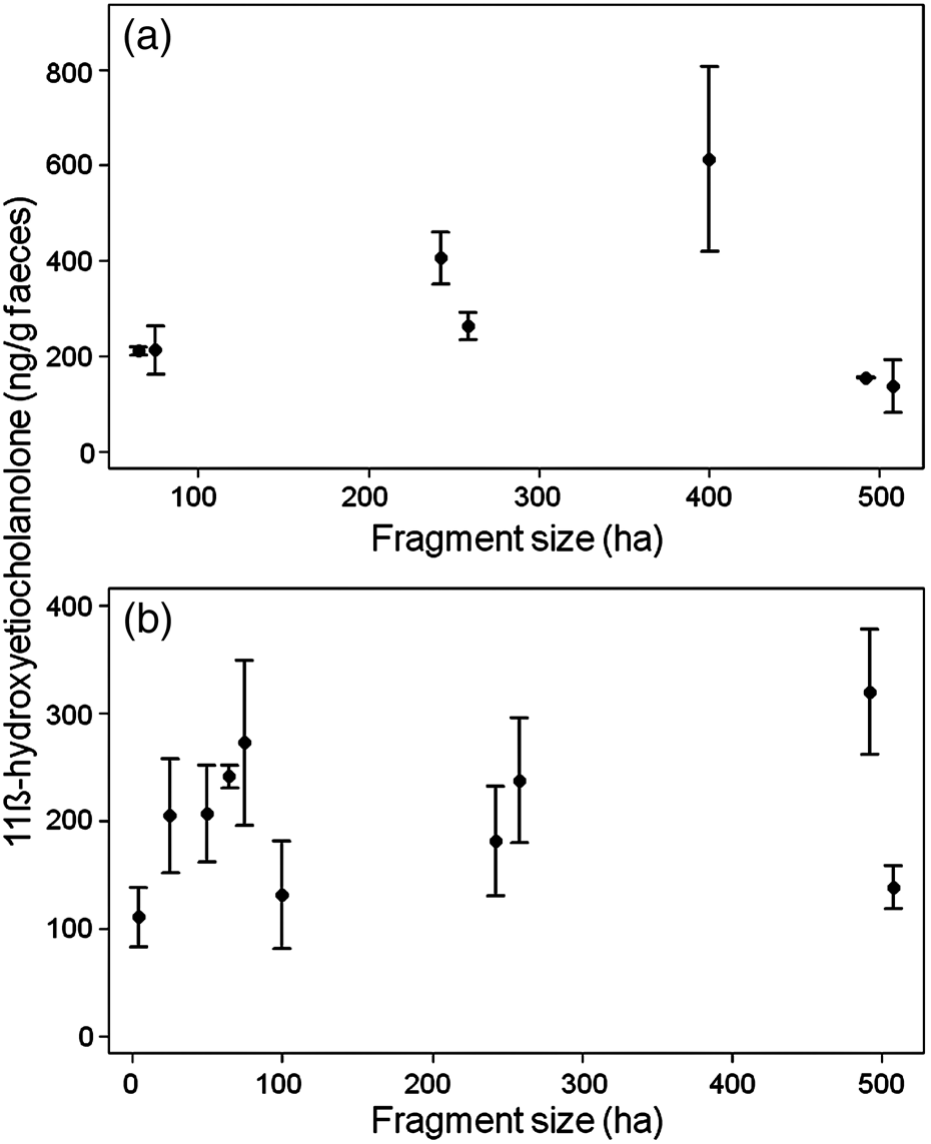
Mean ± SEM faecal glucocorticoid metabolite levels of *Ateles hybridus* (**a**) and *Alouatta seniculus* (**b**) in relationship to forest fragment size.

**Figure 2:**
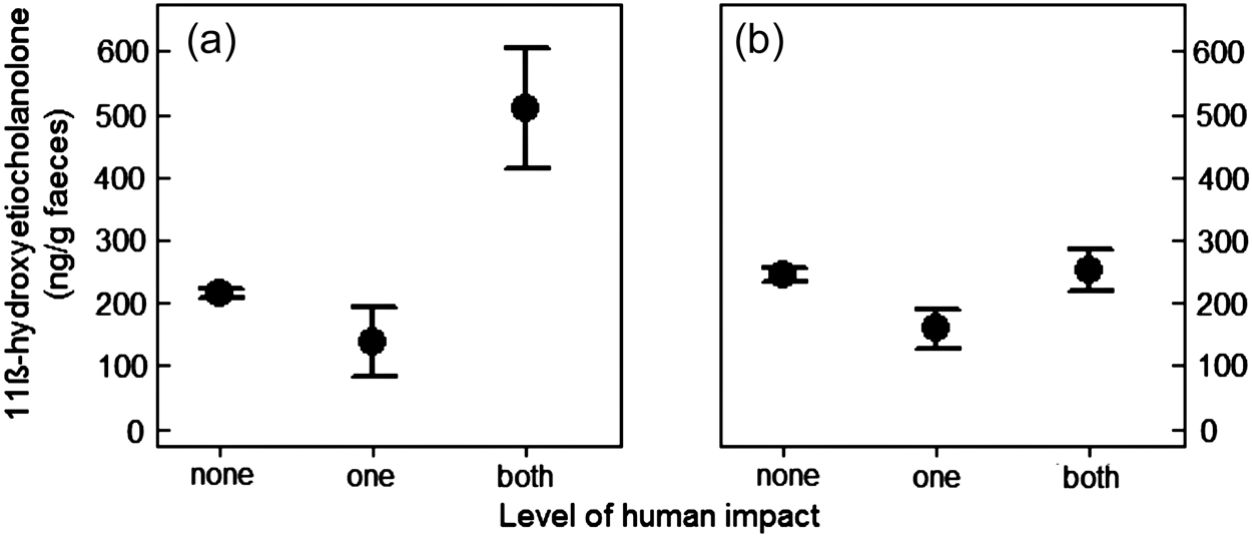
Mean ± SEM faecal glucocorticoid metabolite levels of *Ateles hybridus* (**a**) and *Alouatta seniculus* (**b**) in relationship to level of human impact (none: no hunting or logging, one: either hunting or logging, both: both hunting and logging).

In both species, FGCM levels varied substantially between different fragments (Figure 1). The fragment size did not affect FGCM levels in spider monkeys (*n* = 481 samples; LMM: χ^2^ = 2.06, d.f. = 1, *P* = 0.15; Figure 1a), whereas human impact had an influence (LMM: χ^2^ = 17.5, d.f. = 2, *P* = 0.0001; Figure 2a). Specifically, spider monkeys living in fragments with both kinds of human influence had FGCM levels (mean ± SEM = 496.7 ± 90.0 ng/g) that were more than twice as high as those found in animals living in fragments with no disturbance (mean ± SEM = 206.74 ± 7.5 ng/g; *P*_MCMC_ = 0.01; Table 2) or only one type of disturbance (mean ± SEM = 137.16 ± 45.5 ng/g; *P*_MCMC_ = 0.04). There was no difference between the FGCM levels of spider monkeys living in fragments with no human impact or only one type (*P*_MCMC_ = 0.48, Table 2).

**Table 2:** Results of the linear mixed models examining the influence of forest fragment size and level of human impact on log-transformed faecal glucocorticoid metabolite levels in *Ateles hybridus*

	d.f.	χ^2^	*P* value
Null vs. full model	9	113.38	<0.001*
Variable	Estimate ± SEM	*t*	*P*_MCMC_
Intercept	5.76 ± 0.33	17.52	0.0001*
Fragment size	0.09 ± 0.05	1.81	0.1694
Impact: both–none	−0.93 ± 0.32	−2.88	0.0134*
Impact: both–one	−1.37 ± 0.57	−2.39	0.0400*
Impact: one–none	0.43 ± 0.59	0.72	0.4820
Group size	0.03 ± 0.04	0.83	0.7428
Time	−0.29 ± 0.03	−8.72	0.0001*
Sex	0.14 ± 0.09	1.48	0.1532
Age	−0.33 ± 0.11	−2.84	0.0062*

Overall effect	d.f.	χ^2^	*P*
Female reproductive state	3	21.15	<0.001*
Fragment size	1	2.06	0.1565*
Human impact	2	17.5	0.0001*

*Variables that significantly influenced faecal glucocorticoid metabolite levels.

The results of the second model (comparing fragments with ‘control sites’) were similar to the model using fragment size. The FGCM levels of spider monkeys did not differ between fragments (mean ± SEM = 217.28 ± 8.12 ng/g) and ‘control sites’ (mean ± SEM = 378.51 ± 106.42 ng/g; *n* = 481 samples; LMM, χ^2^ = 0.27, d.f. = 1, *P* = 0.60; Table 3). As in the first model, the level of human impact influenced FGCM levels of spider monkeys (LMM, χ^2^ = 16.21, d.f. = 2, *P* = 0.0003; Table 3).

**Table 3:** Results of the linear mixed models examining the influence of forest type (fragment or ‘control sites’) and level of human impact on log-transformed faecal glucocorticoid metabolite levels in *Ateles hybridus*

	d.f.	χ^2^	*P* value
Null vs. full model	9	72.52	<0.001*
Variable	Estimate ± SEM	*t*	*P*_MCMC_
Intercept	6.33 ± 0.32	19.58	0.0001*
Forest type	−0.20 ± 0.39	−0531	0.5708
Impact: both–none	−1.06 ± 0.34	−3.05	0.0168*
Impact: both–one	1.04 ± 0.57	1.81	0.0868
Impact: one–none	−1.06 ± 0.34	−3.05	0.0172*
Group size	0.10 ± 0.03	2.61	0.3718
Time	−0.23 ± 0.03	−6.30	0.0001*
Sex	−0.01 ± 0.10	−0.15	0.9004
Age	−0.36 ± 0.12	−2.95	0.0030*

Overall effect	d.f.	χ^2^	*P* value
Female reproductive state	3	8.38	0.0386*
Forest type	1	0.27	0.6033*
Human impact	2	16.21	0.0003*

*Variables that significantly influenced faecal glucocorticoid metabolite levels.

In howler monkeys, neither fragment size (Figure 1b) nor human impact (none: mean ± SEM = 245.9 ± 10.6 ng/g; one: 159.4 ± 31.0 ng/g; and both: 251.2 ± 32.9 ng/g; Figure 2b) influenced FGCM levels (*n* = 373 samples, full vs. null model χ^2^ = 13.76, d.f. = 9, *P* = 0.13). The second model showed similar results. Neither human impact nor forest type (fragments: mean ± SEM = 168.61 ± 26.89 ng/g; and ‘control sites’: mean ± SEM = 246.94 ± 10.36 ng/g) influenced FGCM levels of howler monkeys (*n* = 373 samples, full vs. null model, χ^2^ = 11.02, d.f. = 9, *P* = 0.27).

## Discussion

Consistent with our predictions, spider monkeys had higher FGCM levels in fragments with the highest level of human impact compared with less impacted fragments. We did not find such an effect in howler monkeys. In contrast to our predictions, neither fragment size nor forest type (fragments compared to ‘control sites’) influenced FGCM levels of either species. Time of sample collection, age, and female reproductive state influenced FGCM levels in *A. hybridus* (possible explanations are discussed elsewhere; see Rimbach *et al.*, 2013), but we controlled for these factors in data analyses. This study reinforces previous results concerning species-specific differences in the ability to cope with anthropogenic disturbances and strengthens the assumption that brown spider monkeys are more susceptible to human alterations than red howler monkeys.

Proximity to humans, hunting, and logging activities are likely to be perceived as threatening by many animals. Red deer (*Cervus elaphus*) that were chased by humans (Bateson and Bradshaw, 1997) and female African elephants (*Loxodonta africana*) that ranged in areas with high poaching risk (Gobush *et al.*, 2008) had elevated GC levels compared to conspecifics that did not experience the disturbance. The presence of humans can lead to elevated GC levels in several animal taxa (birds: Fowler, 1999; Müllner *et al.*, 2004; Thiel *et al.*, 2011; reptiles: French *et al.*, 2010; mammals: Creel *et al.*, 2002; Barja *et al.*, 2007; Behie *et al.*, 2010; Muehlenbein *et al.*, 2012; Piñeiro *et al.*, 2012; Zwijacz-Kozica *et al.*, 2012). Moreover, proximity to humans can impair the breeding success of animals (Ellenberg *et al.*, 2006; Hinam and St Clair, 2008; Strasser and Heath, 2013), which might be caused by increased GC levels (Ellenberg *et al.*, 2007; Charbonnel *et al.*, 2008). Furthermore, logging activities can result in elevated GC levels (Wasser *et al.*, 1997). Concordant with previous studies, we found elevated FGCM levels of brown spider monkeys in fragments where logging and hunting occurred. Whether this elevation of FGCM levels indicates a state of chronic stress with potential negative consequences on health and fitness is difficult to assess, especially in such long-lived animals, and is beyond the scope of this paper (for a discussion of chronic stress, see e.g. Boonstra, 2013). In contrast to spider monkeys, we did not find an effect of logging and hunting on FGCM levels of red howler monkeys, which suggests that this species might have a lower sensitivity to react to anthropogenic disturbances with an activation of the hypothalamic–pituitary–adrenal axis than brown spider monkeys. Likewise, the intensity of human presence (number of tourists) did not influence the physiological stress levels of another howler monkey species (*Alouatta palliata mexicana*; Aguilar-Melo *et al.*, 2013, but see Behie *et al.*, 2010).

Fruit availability often declines in small and established (>10 years) fragments (Putz *et al.*, 1990; Cordeiro and Howe, 2001; Arroyo-Rodríguez and Mandujano, 2006; Dunn *et al.*, 2010), and low food availability can cause elevated GC levels in primates (Cavigelli, 1999; Muller and Wrangham, 2004; Chapman *et al.*, 2007; Behie *et al.*, 2010). Surprisingly, spider monkeys living in small fragments did not show elevated FGCM levels compared with those living in larger ones, although they potentially experience low levels of food availability and high resource competition. Although some fragments included in this study are very small, drastic changes in food availability might not have occurred yet because most of these fragments have been created rather recently (<10 years). This might explain why fragment size did not influence FGCM levels of either species. Alternatively, it could be that elevated GC levels were associated with low food availability in some fragments, but we are lacking data on resource availability for most fragments and thus, cannot test this assumption. However, two very small fragments (Jamaica and Juntas) were isolated about 100 years ago, and food availability is extremely low (Gómez-Posada *et al.*, 2010). Nevertheless, howler monkeys are able to persist in these fragments and seem to maintain relatively low GC levels.

In contrast to frugivores, folivores are often able to persist in moderately disturbed areas (Johns and Skorupa, 1987), probably because leaf quantity and quality are often higher in disturbed areas, especially at edges, where light exposure is high (Johns, 1988; Ganzhorn, 1995; Irwin, 2008). In the case of howler monkeys, altered leaf availability and quality might compensate for negative effects associated with small fragments and human impact because of their mainly folivorous diet. This supports the notion that they are capable of habituating to human activities, which is likely to explain why we did not find increased FGCM levels in individuals that live in small and disturbed fragments.

The observed inter-specific differences in responsiveness to human impact could also be the result of a different ‘perception’ of stressful factors. Differences in population densities between fragments might be a crucial factor determining GC levels of animals, and howler monkeys have been reported to live at very high densities in fragments (Rumiz, 1990; Ostro *et al.*, 2001; Gómez-Posada *et al.*, 2010; Link *et al.*, 2010). Owing to the lack of data on population densities for most fragments, we were not able to include this variable. Future research should include this factor and thus help to determine whether the species shows variation in FGCM levels according to different population density levels.

Generally, our results support previous findings emphasizing a species-specific effect of human disturbance and habitat fragmentation on adrenocortical activity. This specificity might be the reason for inconsistent results revealed by previous studies that used GC levels as markers of physiological stress in a conservation context (reviewed by Busch and Hayward, 2009). While several studies report elevated GC levels in response to anthropogenic disturbances (Wasser *et al.*, 1997; Lucas *et al.*, 2006; Martínez-Mota *et al.*, 2007; Rangel-Negrín *et al.*, 2009; Janin *et al.*, 2011; Jaimez *et al.*, 2012), others found the reverse, or no effect at all. For instance, red-bellied lemurs (*Eulemur rubriventer*; Tecot, 2008) and African forest elephants (*Loxodonta africana cyclotis*; Munshi-South *et al.*, 2008) show higher GC levels in undisturbed areas than conspecifics in disturbed habitats. Canadian grizzly bears (*Ursus arctos*) exhibit lower GC levels in areas with high poaching activity compared with less disturbed areas (Wasser *et al.*, 2004), whereas Alaskan brown bears (*Ursus arctos horribilis*), a closely related subspecies, show no effect of human presence on GCs (von der Ohe *et al.*, 2004). This demonstrates that species probably differ in their sensitivity to disturbances and that not all species respond with a predicable change in GC levels, or (not mutually exclusive) that such a physiological response depends on the degree of the threat perceived.

One important limitation of our study is the lack of faecal samples from extensive and continuous forests and uneven sample sizes between fragments. The small sample size in fragments where primates are being hunted reflects the challenge of encountering and following arboreal animals that are wary and fearful of humans. Although we have only few samples of *A. hybridus* from two fragments with both types of human impact, the FGCM levels of all those samples are much higher than for those collected in other fragments. Thus, it is conceivable that these differences in FGCM levels reflect true differences, although additional studies should be conducted to confirm these results. Another impeding factor for sample collection is the high degree of fragmentation of the remaining habitat of *A. hybridus* (Urbani *et al.*, 2008) that exacerbated the access to large forests. Our sample size of fragments with only one type of human impact is also small, and future research (with a larger number of fragments) may clarify which of the two factors (logging or hunting) drives the observed elevation in FGCM levels in brown spider monkeys. Moreover, the intensity of human activities is likely to be another important factor influencing FGCM levels. Unfortunately, we were not able to quantify the intensity of hunting and logging activities during this study (e.g. how many people worked in the fragments per day, how many primates were killed in a defined time period). One way to overcome problems concerning the acquisition of data on the intensity of human activities may be to collect samples of the same population before, during, and after logging or hunting activities occur and investigate how FGCM levels change accordingly.

In the absence of data on fitness and health parameters, the results of this study should be interpreted conservatively and they should be confirmed by additional studies. Nevertheless, by controlling for many variables that can potentially confound GC levels (of which some have been neglected previously) and by comparing GC levels of two species that occur (at least partly) in the same fragments, we provide important evidence for species-specific differences in physiological responsiveness and susceptibility to anthropogenic disturbances.

This study reveals that some species (e.g. howler monkeys) may not be negatively influenced by a moderate level of human activity and suggests that agricultural ecosystems could be of use to conserve them. However, our results also demonstrate that GC levels of some species can be elevated in response to anthropogenic disturbances. To what extent these GC elevations reflect a situation of chronic stress with potentially negative fitness consequences or are merely a reflection of an acute adrenocortical reaction to ongoing human activities and, as such, might be adaptive to cope with a short-term challenge (without consequences on fitness) is impossible to assess in the absence of longer-term investigations on fitness and health parameters. It is conceivable, however, that if anthropogenic disturbances persist in the long term, this can potentially lead to a state of chronic stress, which might limit the future viability of animal populations. This study emphasizes the need for the active protection of continuous forests for the conservation of species with low coping abilities (e.g. spider monkeys). Measurements of physiological stress levels should be used to monitor populations living in disturbed areas and to assess the success (concerning amelioration or minimization of stress) of conservation strategies such as corridors connecting fragments and the promotion of alternative sources of animal protein for the human population (e.g. to decrease hunting pressure).
